# Mind the gaps and educational disparities in awareness of cancer risk factors: a cross-sectional study amongst the general public in Sweden

**DOI:** 10.1186/s12889-026-26882-8

**Published:** 2026-03-05

**Authors:** Cecilia Hultstrand, Ellen Brynskog, Andreas Karlsson Rosenblad, Anna-Lena Sunesson, Thomas Björk-Eriksson, Lena Sharp

**Affiliations:** 1 Regional Cancer Centre North, Norrlands University Hospital, Umeå, 901 85 Sweden; 2 Department of Nursing, Umeå University, Umeå, 901 87 Sweden; 3 Regional Cancer Centre West, Sahlgrenska University Hospital, Gothenburg, SE Sweden; 4 Department of Health Sciences, Karlstad University, Karlstad, 651 88 Sweden; 5 Regional Cancer Centre Stockholm-Gotland, Lindhagensgatan 98, Stockholm, 112 18 Sweden; 6 Department of Statistics, Uppsala University, Uppsala, Sweden; 7 Department of Diagnostics and Intervention, Umeå University, 901 87 Umeå, Sweden; 8 Department of Oncology, Institute of Clinical Sciences, Sahlgrenska Academy, University of Gothenburg, Gothenburg, Sweden

**Keywords:** Cancer awareness, Cancer risk factors, Cancer prevention, Joint Action Prevent NCD

## Abstract

**Background:**

Research indicates that about 40% of all cancer cases within the European Union (EU) are preventable. Public awareness of modifiable risk factors is essential for informed health-related decision-making. Systematic assessments of public awareness are crucial for identifying awareness gaps and guiding targeted public health interventions. This study aimed to examine awareness of cancer risk factors among the Swedish general public, and to examine the attitude towards lifestyle changes for cancer prevention.

**Methods:**

This cross-sectional study used a pre-existing data set with a randomly selected sample of 1520 participants (18–84 years old) recruited from a Swedish online survey panel in April 2024. Statistical analyses utilized post-stratification weights to make the results representative for the general Swedish population. Pearson’s χ^2^-test and weighted adjusted logistic regression were used to test for associations between demographic characteristics, believing that changed lifestyle habits could reduce one’s cancer risk, and awareness of 20 established risk factors for cancer.

**Results:**

A majority (63.6%) of the respondents believed that one’s cancer risk could be reduced through changed lifestyle habits. Most were aware of smoking (97.1%), sun exposure (92.4%), hereditary factors (91.0%), sunbeds (90.2%), and air pollution (90.2%), while fewer were aware of alcohol (64.9%), obesity (61.6%), overweight (58.1%), and processed meat (53.3%) as cancer risk factors. A minority of the responders were aware of low levels of physical activity (48.1%), red meat (38.9%), low intake of fruit and vegetables (32.9%), low intake of whole grains (23.7%) and not breast-feeding one’s child (9.3%) as risk factors. For most risk factors, the awareness was significantly higher among college/university educated respondents.

**Conclusions:**

Beside significant awareness gaps among the Swedish general public regarding several established cancer risk factors, this study found an educational gradient, illuminating important differences in cancer prevention awareness. Achieving meaningful improvements in cancer prevention awareness requires coordinated system-level and policy-level actions to reduce the educational gradient and ensure equitable access to information. This could in turn increase people’s ability to make well-informed decisions regarding their lifestyle habits and preventive measures.

## Background

The cancer incidence is continuously increasing both globally [1, 2], and in Sweden [3], placing a significant burden on the people affected, healthcare organizations and societies. It has been estimated that approximately 40% of all cancer cases within the European Union (EU) could be prevented through healthier lifestyles, preventive measures (such as vaccination and screening) as well as through healthier environments and workplaces [4].

Since 1987, the European Code Against Cancer (ECAC) has aimed to raise awareness for modifiable cancer risk factors. The recently updated edition outlines 14 evidence-based recommendations for reducing cancer risk (IARC) [5]. Despite its longevity, awareness about the ECAC has been reported as low. A recent study showed that only 3.7% of the Swedish general public had heard of the ECAC [6].

Despite the general low ECAC awareness, research suggests that public awareness concerning specific cancer risk factors varies. The carcinogenic effects of tobacco have been evident for decades [7] and is commonly recognized by the general public [8–11]. In contrast, several other risk factors (despite being supported by robust epidemiological evidence), remain considerably less recognized. International research finds that people are least aware of the cancer risks concerning alcohol consumption, dietary habits and human papillomavirus (HPV) [8, 9, 11, 12], even if the awareness differs both between and within countries.

Awareness of the cancer risk factors is a significant prerequisite for making well-informed health related decisions and a key aspect of individual autonomy. Thus, it is concerning that previous research finds a discrepancy between the available evidence on cancer risk factors and public awareness. Additionally, previous studies indicate that cancer risk factor awareness is influenced by socioeconomic factors [11, 13].

The body of evidence regarding cancer risk factors and prevention is constantly evolving. In recent years for example, significant scientific progress has been made in understanding the role of both obesity [14–16] and physical inactivity [16]. Consequently, the systematic examination of public awareness of cancer risk factors is a critical component of effective public health strategies. Such research enables health authorities to identify awareness gaps, prioritize resources and design targeted interventions that address the specific needs of diverse population groups. As the field of cancer prevention continues to evolve, it is imperative that public awareness progresses in tandem with these scientific developments to ensure informed decision-making. To the authors’ knowledge, research on public awareness of cancer risk factors in Sweden is both scarce and out-dated.

### Aim

The aim of the present study is to examine awareness of cancer risk factors among the Swedish general public and to examine the attitude towards lifestyle changes for cancer prevention. 

## Methods

### Study design

The present cross-sectional study is part of a larger study on awareness of and attitudes towards the ECAC. A detailed description of the cohort and data collection process is presented in Hultstrand et al. [6].

### Study population

Respondents consist of panellists from an online survey, recruited from *Sverigepanelen* (Sweden Panel), a survey panel operated by the data analysis company Novus™ [17] with approximately 50 000 panellists living in Sweden, aged 18–84 years.

### Questionnaire

An online questionnaire, developed by the research team and described in more detail in a previous publication [6], was used. The questionnaire included 15 items on awareness of risk factors for cancer and attitudes and behaviours related to cancer prevention. The present study examines the results from items focusing on cancer risk factor awareness.

### Data collection

The data were collected between the 2nd and the 11th of April, 2024. In total, 3099 panellists were consecutively recruited, until the intended goal of 1520 panellists had accepted to participate and completed the questionnaire (response rate 49.0%). The sample size was chosen to enable valid sub-analyses. Respondents could not alter registered responses. The questionnaire was programmed and administrated to the panel by Novus.

### Study variables

#### Outcomes

The respondents’ beliefs that changed lifestyle habits could reduce risk and awareness of cancer risk factors were measured using the following two questionnaire items:

1. “I believe that people can reduce their risk of getting cancer by changing their lifestyle habits”
2. “Would you say that the following factors increase the risk of developing cancer?”

Item # 1 had Likert scale response options (1–5 points), with 1 point labelled as “Do not agree at all” and 5 points as “Totally agree”, in addition to the “Do not know” option. For these items, responses at 4–5 points were categorized as “Yes”, while all other answers were categorized as “No”.

Item # 2 presented risk factors with strong evidence, risk factors with limited evidence and myths. Risk factors were categorized based on the ECAC (5th ed.), as well as the Word Cancer Research Fund's recommendations [18] and assessment of current available evidence [19, 20]. Consequently, strong evidence is considered to be “*evidence strong enough to support a judgment of a convincing or probable casual relationship and generally justify making recommendations*” (p. 40) [20].

For the scope of this study, the following 20 risk factors are included in the analyses: smoking; second-hand smoking; heredity factors; sun exposure; sunbeds; air pollution; radon; alcohol; obesity; overweight; hormone therapy; processed meat; low level of physical activity; red meat; low intake of fruit; low fibre intake; viral and bacterial infections; sedentary; low intake of whole grains and not breastfeeding your child. The following response options were available: “Yes”, “No”, “Do not know”, which were dichotomized as “Yes” and “No” (with “Do not know” responses included in the “No” category).

#### Predictors

The demographic characteristics collected from the study sample included gender, age (years), national background, highest completed level of education, personal income measured as Swedish Krona (SEK; €1 ≈ 10 SEK) per month, marital status, and geographic area of residence. Further details are provided in a previous publication [6]. Gender had the following three response options: Male, Female, and Other. However, since no “Other” responses were provided, gender was dichotomized as Male/Female. National background was dichotomized as Swedish/Foreign. Income levels were categorized into the following three groups; “ < 20,000 SEK/month or Other”, “20,000–39,999 SEK/month”, and “ ≥ 40,000 SEK/month”, with those answering No income classified as < 20,000 SEK/month and those responding *Don’t know* or *Does not want to disclose* classified as Other. Education levels were measured using the three main categories of the Swedish education system; Primary school (G*rundskola* or equivalent), Secondary school (*Gymnasium* or equivalent), or College/university (*Högskola/universitet*). In addition, the respondents had the possibility to select the response option “None completed”, although none of the respondents selected this alternative. For the present study, education level was dichotomized as College/University education (Yes/No). Marital status had the five following response categories; Married, Cohabiting, Living alone, Partnership, Living with parents, and Other. For the present study, these five categories were dichotomized as Living alone (Yes/No), with those giving their marital status as “Living alone” classified as Yes and all the other classified as No. Finally, the respondents’ geographic areas of residence were classified into the following six Swedish healthcare regions (HCRs); Stockholm-Gotland, Mid-Sweden, Southeast, East, South, West, and North.

### Statistical analyses

Unless otherwise specified, the statistical analyses utilized post-stratification weights to make the results representative for the general Swedish population. As described in our previously published study, the post-stratification (made in order to adjust for possible biases in the sample compared to the target population) was performed with regards to gender, age, education, and expressed political party orientation [6].

Categorical data are presented as frequencies and percentages, n (%), while continuous data are given as mean values with accompanying standard deviations (SDs). Tests of differences due to demographic characteristics were performed using Pearson’s χ^2^-test with Rao-Scott second-order corrections, with *P*-values calculated using a Satterthwaite approximation to the distribution and denominator degrees of freedom according to Thomas and Rao [21]. For this purpose, age was categorized as 18–34, 35–49, 50–64, and 65–84 years old.

The magnitudes of the associations between demographic characteristics (predictors) and the outcomes (believing that the risk of getting cancer may be reduced by changed lifestyle habits and awareness of the 20 cancer risk factors) were estimated using weighted adjusted logistic regression models, calculated using generalised linear models with a quasi-binomial family and a logit link function. Together with inverse-probability weighting and design-based standard errors, separately for the 20 risk factors. For all models, predictors were included simultaneously as independent variables, with age (years) as a continuous variable. Female as reference category for gender. Foreign/Other as reference category for national background. No as reference category for college/university education. “ < 20,000/Other” as reference category for income. No as reference category for living alone and Stockholm-Gotland as reference category for HCR. The results are reported as adjusted odds ratios (AORs) with 95% confidence intervals (CIs).

All statistical analyses were performed using R 4.3.1 or higher (R Foundation for Statistical Computing, Vienna, Austria) together with the R package survey [22] with two-sided *P*-values < 0.05 were considered statistically significant.

## Results

### Demographics

The demographic characteristics, with weighted and unweighted distributions among the 1520 participants, are presented in Table 1. We found a slight difference in gender, with 50.5% men and 49.5% women among the respondents. Younger persons (< 50 years old) were somewhat underrepresented in the sample, in particular those 18–34 years old, which constituted only 18.9% of the respondents and were thus up-weighted to 28.1%. Conversely, those aged ≥ 50 years were somewhat overrepresented and thus had to be down-weighted. This in particular affected those aged 65–84 years old, who were down-weighted from 30.5% to 23.4%.

**Table 1:** Demographic characteristics with weighted and unweighted distributions among the 1520 participants in the present study

	Unweighted		Weighted	
Variable	n	%	n	%
Gender				
Male	767	50.5	768	50.5
Female	753	49.5	752	49.5
Age (years)^a^				
18–34	288	18.9	428	28.1
35–49	362	23.8	376	24.7
50–64	407	26.8	361	23.8
65–84	463	30.5	355	23.4
National background				
Swedish	1243	81.8	1216	80.0
Foreign	102	6.7	99	6.5
Missing	175	11.5	205	13.5
Education level				
Primary school	65	4.3	114	7.5
Secondary school	506	33.3	812	53.4
College/University	949	62.4	594	39.1
Income (SEK/month)				
< 20,000	323	21.2	427	28.1
20,000–39,999	595	39.1	598	39.3
≥ 40,000	538	35.4	433	28.5
Don’t know/Does not want to disclose	64	4.2	62	4.1
Living alone				
Yes	366	24.1	378	24.8
No	1154	75.9	1142	75.2
Swedish Health Care Region				
Stockholm-Gotland	393	25.9	373.1	24.5
Mid-Sweden	312	20.5	314.6	20.7
Southeast	135	8.9	148.8	9.8
South	253	16.6	277.1	18.2
West	274	18.0	274.4	18.1
North	153	10.1	131.9	8.7

*SD* Standard deviation, *SEK* Swedish Krona. 10,000 SEK ≈ €885

^a^The unweighted mean (SD) age was 53.3 (17.5) years, while the weighted mean (SD) age was 48.7 (18.5) years

In total, 81.8% of the participants reported having a Swedish background, a slight overrepresentation that resulted in a down-weight to 80.0%. Participants with a college/university education were heavily over-represented at 62.4%, resulting in a down-weight to 39.1%, while those with a secondary school education level were up-weighted from 33.3% to 53.4%. Income level was somewhat more representative among the respondents, with an up-weighting of those having an income of < 20,000 SEK/month from 21.2% to 28.1% and a down-weighting of those having an income of ≥ 40,000 SEK/month from 35.4% to 28.5%. Living alone was reported by 24.1% of the respondents, which was quite representative compared to the general population, and thus resulting in only a small up-weighting to 24.8%. Geographic area of residence was likewise quite representative, resulting in only minor up- and down-weightings.

### Beliefs about cancer risk and changes in lifestyle habits

Results from the weighted adjusted logistic regression analyses of the associations between demographic characteristics and the belief that cancer risks may be reduced by changed lifestyle habits are provided in Table 2. Significant associations were found for gender, age, education level and HCR, with men being 1.91 times more likely to have this belief, compared to women (*P* < 0.001). Older respondents were somewhat less likely to have this belief (AOR 0.99 per each additional year of age; *P* = 0.022). College/university educated respondents were 1.98 times more likely to have this belief compared to those without such education (*P* < 0.001). Finally, those living in the Southeast HCR were 1.91 time more likely to believe that cancer risks may be reduced by changed lifestyle habits, compared to those living in the Stockholm-Gotland HCR (*P* = 0.015).

**Table 2:** Results from weighted adjusted logistic regression analyses of the association between demographic characteristics and believing that the risk of getting cancer may be reduced by changed lifestyle habits

	Frequencies and percentages			Regression model		
Variable	n	%	*P*-value^a^	AOR	95% CI	*P*-value
Total	966	63.6	N/A			
Gender			** < 0.001**			
Male	533	69.5		1.91	1.44–2.54	** < 0.001**
Female	433	57.5		Ref		
Age (years)			**0.006**	0.99	0.98–1.00	**0.022**
18–34	308	72.2				
35–49	217	57.8				
50–64	220	61.0				
65–84	220	62.0				
National background			0.815			
Swedish	775	63.8		1.06	0.74–1.51	0.760
Foreign/Missing	191	62.8		Ref		
College/University education			** < 0.001**			
Yes	418	70.4		1.98	1.51–2.60	** < 0.001**
No	548	59.2		Ref		
Income (SEK/month)			0.571			
< 20,000/Other^b^	299	61.2		Ref		
20,000–39,999	386	64.6		1.04	0.74–1.46	0.822
≥ 40,000	281	64.9		0.79	0.55–1.14	0.212
Living alone			0.639			
Yes	245	64.8		1.12	0.82–1.53	0.463
No	721	63.1		Ref		
Swedish Health Care Region			0.308			
Stockholm-Gotland	231	61.9		Ref		
Mid-Sweden	187	59.6		0.93	0.63–1.38	0.720
Southeast	110	73.8		1.91	1.13–3.22	**0.015**
South	178	64.2		1.11	0.73–1.70	0.623
West	174	63.6		1.13	0.76–1.68	0.541
North	85	64.8		1.11	0.68–1.81	0.675

Participants were classified as agreeing on the statement *I believe that people could reduce their risk of getting cancer by changing their lifestyle habits* if they answered 4 or 5 on a scale of 1–5 points, where a value of 5 points was stated to mean *Agree completely* and a value of 1 point was stated to mean *Disagree completely*. Those answering 1–3 or *Don’t know* were classified as not agreeing. Frequencies and percentages are estimated based on post-stratification weights and then rounded, meaning that not all numbers may add up due to rounding errors. Significant *P*-values are given in **bold**

*AOR* Adjusted odds ratio, *CI* Confidence interval, *Ref.* Reference category, *SEK* Swedish Krona (10,000 SEK ≈ €885)

^a^Calculated using Pearson’s χ^2^-statistic with the Rao-Scott second-order correction

^b^Including *Don’t know and Does not want to disclose*

### Awareness of risk factors for cancer for different demographic groups—univariate results

Awareness of the 20 cancer risk factors according to the weighted distribution of the demographic characteristics are provided in Tables 3, 4, 5 and 6. It should be noted that the awareness of smoking, second-hand smoking, hereditary factors, sun exposure, sunbeds, air pollution and radon was overall high (with the overall awareness being > 85%). Awareness was between 50 and 65%, for alcohol, obesity, overweight, and processed meat. In contrast, the awareness of low intake of whole grains and not breastfeeding was lower, with the overall awareness being < 25% for both risk factors.

**Table 3:** Awareness of smoking, secondhand smoking, hereditary factors, sun exposure, and sunbeds as risk factors for cancer according to the weighted distribution of the demographic characteristics

	Smoking			Secondhand smoking			Hereditary factors			Sun exposure			Sunbeds		
Variable	n	%	*P*-value	n	%	*P*-value	n	%	*P*-value	n	%	*P*-value	n	%	*P*-value
Total	1476	97.1	N/A	1306	85.9	N/A	1383	91.0	N/A	1404	92.4	N/A	1371	90.2	N/A
Gender			0.524			0.878			0.275			0.651			**0.005**
Male	743	96.8		658	85.7		690	89.8		713	92.8		670	87.3	
Female	733	97.5		647	86.1		693	92.2		691	91.9		701	93.2	
Age (years)			0.933			0.549			0.881			0.258			0.581
18–34	415	97.2		362	84.8		394	92.2		389	90.9		382	89.4	
35–49	366	97.4		330	87.6		342	91.0		344	91.4		337	89.6	
50–64	349	96.5		316	87.4		327	90.4		332	91.9		323	89.3	
65–84	346	97.4		298	83.9		320	90.1		339	95.6		329	92.8	
National background			0.424			0.184			0.784			0.675			**0.024**
Swedish	1179	96.9		1036	85.2		1105	90.8		1126	92.6		1085	89.2	
Foreign/Missing	298	98.0		270	88.7		279	91.7		278	91.5		286	94.2	
College/University education			**0.012**			**0.012**			** < 0.001**			**0.012**			**0.001**
Yes	586	98.6		529	89.0		567	95.3		563	94.8		557	93.7	
No	891	96.2		777	83.9		817	88.2		841	90.8		815	88.0	
Income (SEK/month)			0.747			0.772			**0.027**			0.453			0.549
< 20,000/Other^a^	477	97.4		426	87.1		426	87.1		449	91.9		433	88.6	
20,000–39,999	577	96.6		510	85.4		553	92.5		546	91.5		545	91.3	
≥ 40,000	423	97.5		370	85.3		405	93.4		408	94.2		393	90.6	
Living alone			0.524			0.670			0.211			0.141			0.415
Yes	364	96.5		321	85.1		335	88.7		340	90.0		346	91.7	
No	1112	97.3		984	86.2		1048	91.8		1064	93.2		1025	89.7	
Swedish Health Care Region			0.817			0.746			0.376			0.190			0.310
Stockholm-Gotland	363	97.3		330	88.3		342	91.8		349	93.6		342	91.6	
Mid-Sweden	303	96.5		261	82.9		277	88.0		282	89.6		275	87.3	
Southeast	145	97.4		128	86.2		141	94.9		135	90.9		126	84.6	
South	267	96.3		236	85.1		245	88.4		254	91.5		255	92.2	
West	271	98.7		236	86.1		255	92.8		266	96.9		253	92.1	
North	127	96.5		115	87.1		123	93.1		118	89.6		121	91.5	

Frequencies and percentages are estimated based on post-stratification weights and then rounded, meaning that not all numbers may add up due to rounding errors. *P*-values are calculated using Pearson’s χ^2^-statistic with the Rao-Scott second-order correction. Significant *P*-values are given in **bold**

*SEK* Swedish Krona (10,000 SEK ≈ €885)

^a^ Including *Don’t know* and *Does not want to disclose*

**Table 4:** Awareness of air pollution, radon, alcohol, obesity, and overweight as risk factors for cancer according to the weighted distribution of the demographic characteristics

	Air pollution			Radon			Alcohol			Obesity			Overweight		
Variable	n	%	*P*-value	n	%	*P*-value	n	%	*P*-value	n	%	*P*-value	n	%	*P*-value
Total	1371	90.2	N/A	1303	85.7	N/A	986	64.9	N/A	937	61.6	N/A	883	58.1	N/A
Gender			0.164			0.134			**0.004**			0.208			0.084
– Male	703	91.5		644	83.8		464	60.5		488	63.5		467	60.8	
– Female	668	88.8		659	87.6		522	69.4		449	59.7		416	55.3	
Age (years)			0.242			0.064			0.559			** < 0.001**			** < 0.001**
– 18–34	384	89.8		349	81.7		288	67.5		311	72.7		290	67.8	
– 35–49	329	87.5		316	84.1		237	62.9		227	60.5		220	58.5	
– 50–64	327	90.6		325	89.9		226	62.5		200	55.3		189	52.3	
– 65–84	331	93.1		312	87.9		236	66.4		198	55.9		184	51.7	
National background			0.253			0.837			0.905			0.202			0.207
– Swedish	1090	89.7		1041	85.6		788	64.8		737	60.6		693	57.0	
– Foreign/Missing	280	92.3		262	86.3		198	65.3		200	65.7		189	62.3	
College/University education			0.433			** < 0.001**			** < 0.001**			0.234			0.214
– Yes	541	91.0		541	91.0		419	70.6		378	63.7		358	60.3	
– No	830	89.6		762	82.3		567	61.3		558	60.3		524	56.6	
Income (SEK/month)			0.865			0.086			0.735			0.821			0.609
– < 20,000/Other^a^	437	89.4		402	82.3		320	65.4		297	60.7		280	57.3	
– 20,000–39,999	541	90.5		514	85.9		393	65.8		367	61.4		340	56.9	
– ≥ 40,000	393	90.6		387	89.3		273	63.0		273	63.1		262	60.5	
Living alone			0.759			0.623			0.467			0.617			0.976
– Yes	339	89.7		320	84.7		238	62.9		228	60.3		219	58.0	
– No	1032	90.4		983	86.0		749	65.5		709	62.1		664	58.1	
Swedish Health Care Region			**0.032**			0.130			**0.026**			0.231			0.572
– Stockholm-Gotland	336	90.2		321	86.0		244	65.3		216	58.0		215	57.7	
– Mid-Sweden	265	84.2		253	80.3		188	59.8		192	61.1		169	53.6	
– Southeast	140	94.4		137	92.0		116	77.9		101	68.0		92	61.9	
– South	257	92.7		235	84.8		164	59.2		184	66.3		170	61.3	
– West	252	91.9		237	86.4		184	66.9		157	57.2		155	56.3	
– North	120	91.0		120	91.2		91	69.0		86	65.4		82	62.3	

Frequencies and percentages are estimated based on post-stratification weights and then rounded, meaning that not all numbers may add up due to rounding errors. *P*-values are calculated using Pearson’s χ^2^-statistic with the Rao-Scott second-order correction. Significant *P*-values are given in **bold**

*SEK* Swedish Krona (10,000 SEK ≈ €885)

^a^ Including *Don’t know* and *Does not want to disclose*

**Table 5:** Awareness of hormone therapy, processed meat, low level of physical activity, red meat, and low intake of fruit and vegetables as risk factors for cancer according to the weighted distribution of the demographic characteristics

	Hormone therapy			Processed meat			Low level of physical activity			Red meat			Low intake of fruit and vegetables		
Variable	n	%	*P*-value	n	%	*P*-value	n	%	*P*-value	n	%	*P*-value	n	%	*P*-value
Total	676	44.5	N/A	810	53.3	N/A	731	48.1	N/A	592	38.9	N/A	500	32.9	N/A
Gender			**0.027**			**0.047**			0.299			0.141			0.282
Male	315	41.0		384	50.1		382	49.8		282	36.7		240	31.3	
Female	361	48.0		425	56.6		349	46.4		310	41.2		260	34.5	
Age (years)			**0.034**			0.263			0.135			0.129			0.133
18–34	167	39.1		231	54.1		226	52.9		171	40.0		126	29.4	
35–49	183	48.6		217	57.7		188	49.9		164	43.6		125	33.4	
50–64	180	49.9		189	52.2		159	43.9		120	33.3		111	30.8	
65–84	146	41.2		173	48.6		159	44.7		136	38.4		138	38.8	
National background			0.349			0.239			0.854			0.607			0.516
Swedish	551	45.3		635	52.2		583	48.0		468	38.5		394	32.4	
Foreign/Missing	126	41.3		174	57.4		148	48.7		123	40.6		106	35.0	
College/University education			** < 0.001**			** < 0.001**			** < 0.001**			** < 0.001**			** < 0.001**
Yes	306	51.5		386	65.0		328	55.1		307	51.6		234	39.4	
No	370	40.0		423	45.7		404	43.6		285	30.8		266	28.7	
Income (SEK/month)			**0.002**			**0.039**			0.186			0.064			0.309
< 20,000/Other^a^	180	36.9		236	48.2		214	43.8		171	34.9		145	29.6	
20,000–39,999	276	46.2		319	53.4		298	49.9		230	38.4		203	34.0	
≥ 40,000	220	50.7		255	58.8		219	50.5		191	44.2		152	35.1	
Living alone			0.425			0.934			0.186			0.554			0.820
Yes	160	42.3		202	53.5		168	44.4		153	40.5		126	33.5	
No	516	45.2		608	53.2		563	49.3		439	38.4		374	32.7	
Swedish Health Care Region			0.431			0.325			0.719			**0.038**			0.548
Stockholm-Gotland	164	43.8		220	58.9		183	49.0		173	46.3		121	32.5	
Mid-Sweden	130	41.5		152	48.5		142	45.2		104	33.2		93	29.6	
Southeast	80	53.5		78	52.2		80	53.6		51	34.5		52	34.9	
South	118	42.7		140	50.5		127	45.7		95	34.4		87	31.4	
West	120	43.6		152	55.4		131	47.7		113	41.2		105	38.4	
North	64	48.9		68	51.3		69	52.3		55	41.5		41	31.2	

Frequencies and percentages are estimated based on post-stratification weights and then rounded, meaning that not all numbers may add up due to rounding errors. *P*-values are calculated using Pearson’s χ^2^-statistic with the Rao-Scott second-order correction. Significant *P*-values are given in **bold**

*SEK* Swedish Krona (10,000 SEK ≈ €885)

^a^Including *Don’t know* and *Does not want to disclose*

**Table 6:** Awareness of low fibre intake, viral and bacterial infections, sedentary, low intake of whole grains, and not breastfeeding one’s child as risk factors for cancer according to the weighted distribution of the demographic characteristics

	Low fibre intake			Viral and bacterial infections			Sedentary			Low intake of whole grains			Not breastfeeding one’s child		
Variable	n	%	*P*-value	n	%	*P*-value	n	%	*P*-value	n	%	*P*-value	n	%	*P*-value
Total	486	32.0	N/A	489	32.2	N/A	649	42.7	N/A	360	23.7	N/A	141	9.3	N/A
Gender			**0.042**			0.069			0.803			**0.006**			0.721
Male	223	29.1		268	34.9		331	43.1		155	20.2		74	9.6	
Female	262	34.9		221	29.4		318	42.3		205	27.2		67	8.9	
Age (years)			**0.001**			0.379			0.603			**0.023**			0.430
18–34	103	24.2		152	35.6		182	42.6		75	17.5		40	9.3	
35–49	119	31.7		124	32.9		167	44.5		91	24.2		43	11.6	
50–64	119	32.9		104	28.8		142	39.2		98	27.2		30	8.4	
65–84	144	40.6		109	30.8		158	44.4		96	27.1		27	7.7	
National background			0.964			0.994			0.059			0.672			0.369
Swedish	389	32.0		392	32.2		499	41.1		292	24.0		108	8.8	
Foreign/Missing	97	31.8		98	32.2		149	49.1		68	22.5		33	11.0	
College/University education			** < 0.001**			** < 0.001**			**0.001**			** < 0.001**			**0.011**
Yes	250	42.1		232	39.1		288	48.5		177	29.9		72	12.1	
No	236	25.5		257	27.8		360	38.9		183	19.7		69	7.5	
Income (SEK/month)			**0.016**			**0.011**			0.198			0.211			0.217
< 20,000/Other^a^	128	26.3		130	26.6		188	38.5		104	21.3		35	7.2	
20,000–39,999	198	33.1		193	32.3		266	44.5		138	23.1		57	9.5	
≥ 40,000	160	36.8		167	38.5		195	44.9		118	27.1		49	11.3	
Living alone			0.254			0.113			0.137			0.241			0.418
Yes	132	34.9		137	36.3		146	38.6		100	26.5		30	8.1	
No	354	31.0		352	30.8		503	44.0		260	22.8		111	9.7	
Swedish Health Care Region			0.286			0.680			0.678			0.109			0.451
Stockholm-Gotland	118	31.6		120	32.3		154	41.3		78	20.9		27	7.3	
Mid-Sweden	91	28.8		96	30.6		135	42.9		69	21.9		30	9.5	
Southeast	59	39.7		53	35.5		67	45.3		45	30.3		21	13.9	
South	85	30.6		78	28.0		106	38.1		61	22.1		21	7.7	
West	98	35.6		95	34.6		125	45.5		81	29.5		29	10.5	
North	36	27.0		47	35.8		62	46.9		26	19.4		13	9.7	

Frequencies and percentages are estimated based on post-stratification weights and then rounded, meaning that not all numbers may add up due to rounding errors. *P*-values are calculated using Pearson’s χ^2^-statistic with the Rao-Scott second-order correction. Significant *P*-values are given in **bold**.

*SEK* Swedish Krona (10,000 SEK ≈ €885)

^a^ Including *Don’t know* and *Does not want to disclose*

**Fig. 1:**
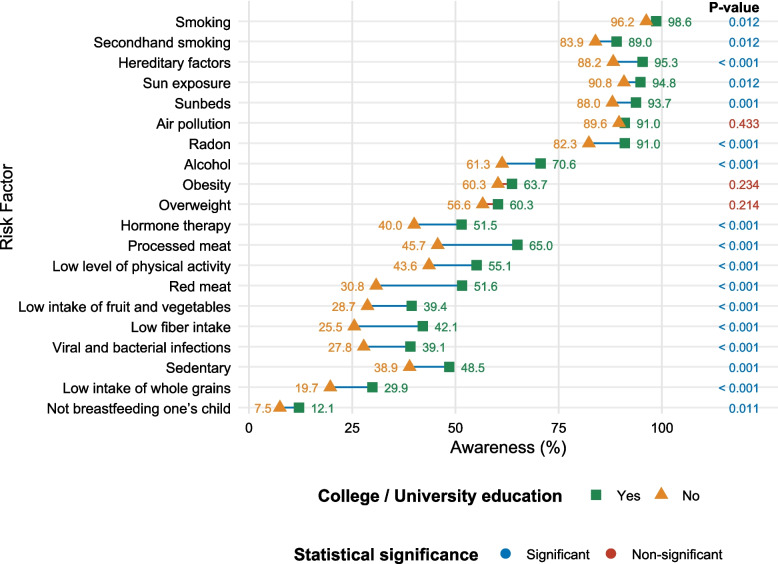
Awareness of the 20 risk factors for cancer according to education level

Having a college/university education was the demographic characteristic most commonly associated with a significantly higher cancer risk factor awareness, with a significant association observed for 17 of the 20 risk factors in the univariate analyses. No significant association was found for air pollution, overweight, and obesity (Fig. 1).

Among the other characteristics (Tables 3, 4, 5 and 6), gender was significantly associated with awareness for six risk factors, in all cases with women having a higher awareness than men. Age was significantly associated with awareness for five risk factors. We found notably higher awareness regarding obesity and overweight among younger participants (aged < 50 years old), higher awareness of hormone therapy among middle-aged participants (aged 36–64 years old), as well as for low fibre intake among the oldest participants (aged 65–84 years old). We also found lower awareness regarding low intake of whole grains, among the youngest age group (18–34 years old).

Income level was significantly associated with awareness of hereditary factors (*P* = 0.027), hormone therapy (*P* = 0.002), processed meat (*P* = 0.039), low fibre intake (*P* = 0.016), and viral and bacterial infections (*P* = 0.011). Participants with higher reported income also showed higher levels of awareness. National background was only significantly associated with awareness of one cancer risk factor (sunbeds) (*P* = 0.024), with a lower awareness observed among those with a Swedish background (Tables 3, 5 and 6).

**Fig. 2:**
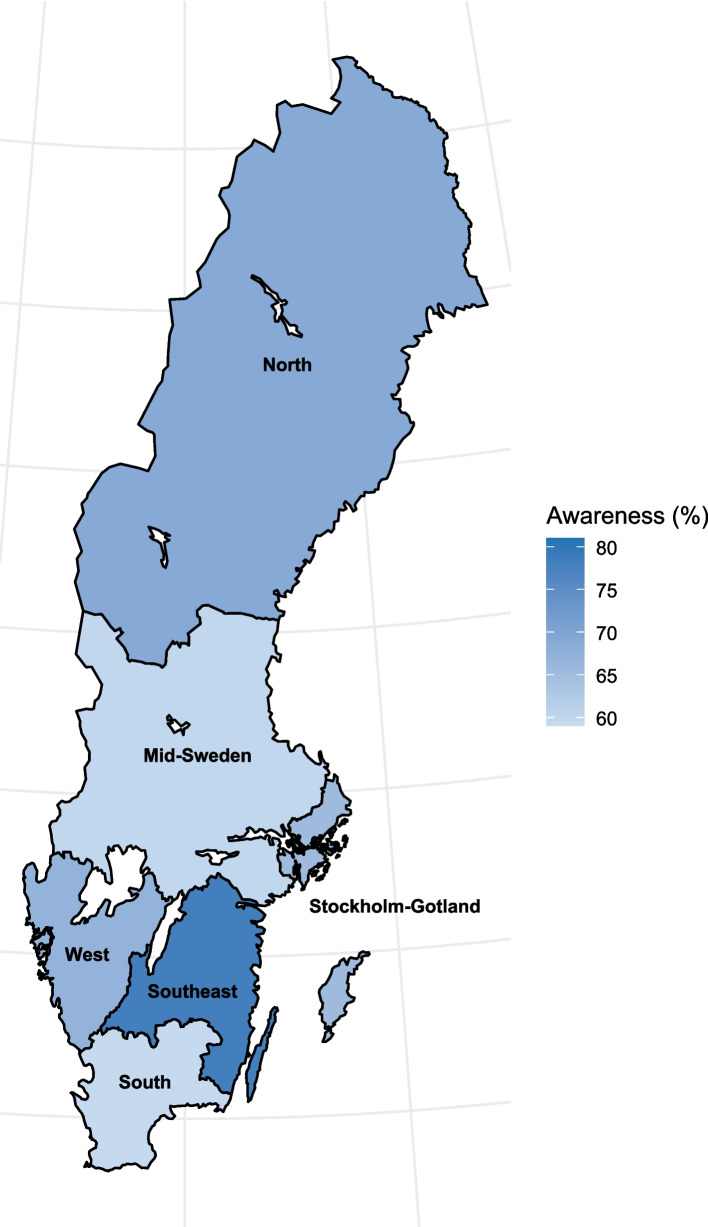
Awareness of alcohol as a risk factor for cancer in the six Swedish health care regions

Finally, as shown in Tables 4 and 5, HCR was significantly associated with awareness of air pollution (*P* = 0.032), alcohol (*P* = 0.026) and red meat (*P* = 0.038). The highest awareness of air pollution and alcohol was observed among respondents from the Southeast HCR, while the Stockholm-Gotland HCR had the highest awareness of red meat. Notably, for alcohol, the difference in awareness between respondents in the Southeast HCR (77.9%) and the South HCR (59.2%) was 18.7 percentage points (Fig. 2).

### Associations between demographic characteristics and awareness of risk factors for cancer—regression results

**Fig. 3:**
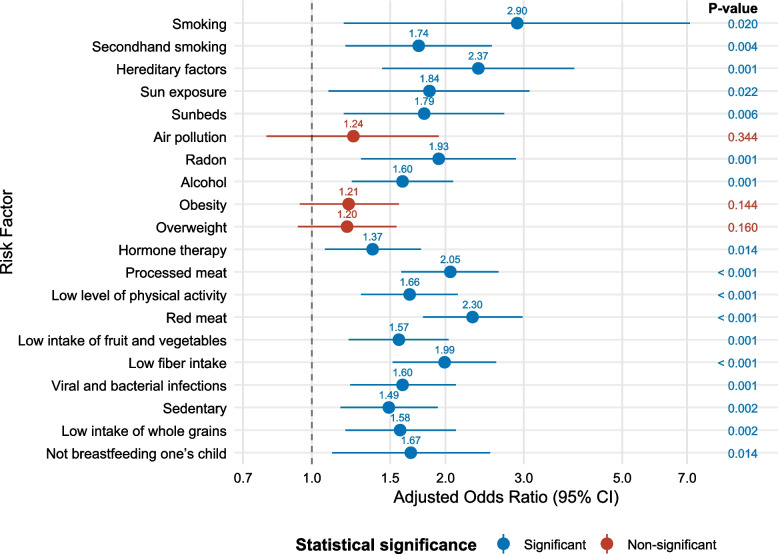
Association between having a college/university education, compared to not having a college/university education, and awareness of the 20 risk factors for cancer

Results from the weighted adjusted logistic regression analyses of the association between demographic characteristics and awareness of the 20 cancer risk factors are provided in Tables 7, 8, 9 and 10. Again, having a college/university education was significantly associated with higher awareness for 17 cancer risk factors (Fig. 3).

**Table 7:** Results from weighted adjusted logistic regression analyses of the association between demographic characteristics and awareness of smoking, secondhand smoking, hereditary factors, sun exposure, and sunbeds as risk factors for cancer

	Smoking			Secondhand smoking			Hereditary factors			Sun exposure			Sunbeds		
Variable	AOR	95% CI	*P*-value	AOR	95% CI	*P*-value	AOR	95% CI	*P*-value	AOR	95% CI	*P*-value	AOR	95% CI	*P*-value
Male gender	0.92	0.40–2.16	0.855	1.11	0.75–1.64	0.596	0.73	0.42–1.25	0.251	1.23	0.73–2.07	0.435	0.50	0.30–0.84	**0.008**
Age (years)	1.00	0.98–1.02	0.938	1.00	0.99–1.01	0.795	1.00	0.98–1.01	0.576	1.01	1.00–1.03	0.069	1.01	0.99–1.02	0.262
Swedish background	0.65	0.22–1.91	0.434	0.73	0.45–1.19	0.209	0.87	0.42–1.82	0.710	1.12	0.58–2.18	0.732	0.51	0.27–0.95	**0.035**
College/University education	2.90	1.18–7.10	**0.020**	1.74	1.19–2.54	**0.004**	2.37	1.44–3.90	**0.001**	1.84	1.09–3.09	**0.022**	1.79	1.18–2.71	**0.006**
Income (SEK/month)															
< 20,000/Other^a^	Ref			Ref			Ref			Ref			Ref		
20,000–39,999	0.67	0.25–1.84	0.440	0.80	0.49–1.29	0.356	1.80	1.00–3.24	**0.049**	0.78	0.42–1.48	0.449	1.35	0.76–2.39	0.301
≥ 40,000	0.66	0.19–2.33	0.522	0.64	0.38–1.09	0.104	1.69	0.88–3.23	0.113	0.88	0.43–1.77	0.715	1.16	0.62–2.20	0.641
Living alone	0.75	0.28–1.97	0.556	0.88	0.58–1.32	0.532	0.75	0.44–1.29	0.300	0.64	0.37–1.11	0.112	1.26	0.70–2.28	0.436
Swedish Health Care Region															
Stockholm-Gotland	Ref			Ref			Ref			Ref			Ref		
Mid-Sweden	0.80	0.28–2.29	0.673	0.65	0.37–1.12	0.117	0.70	0.34–1.42	0.321	0.62	0.30–1.26	0.186	0.67	0.34–1.30	0.233
Southeast	1.12	0.23–5.46	0.889	0.85	0.39–1.87	0.692	1.97	0.66–5.91	0.224	0.73	0.29–1.83	0.500	0.58	0.25–1.36	0.213
South	0.72	0.23–2.23	0.567	0.74	0.42–1.29	0.283	0.76	0.36–1.63	0.483	0.77	0.32–1.82	0.551	1.18	0.55–2.54	0.664
West	2.06	0.50–8.49	0.319	0.80	0.46–1.39	0.430	1.24	0.57–2.72	0.588	2.10	0.82–5.38	0.122	1.12	0.49–2.57	0.783
North	0.77	0.16–3.65	0.739	0.88	0.42–1.82	0.728	1.31	0.44–3.88	0.628	0.67	0.26–1.69	0.391	1.06	0.42–2.66	0.907

The regression models included all variables in the table. Significant *P*-values are given in **bold**

*AOR* Adjusted odds ratio, *CI* Confidence interval, *Ref*. Reference category, *SEK* Swedish Krona (10,000 SEK ≈ €885)

^a^ Including *Don’t know* and *Does not want to disclose*

**Table 8:** Results from weighted adjusted logistic regression analyses of the association between demographic characteristics and awareness of air pollution, radon, alcohol, obesity, and overweight as risk factors for cancer

	Air pollution			Radon			Alcohol			Obesity			Overweight		
Variable	AOR	95% CI	*P*-value	AOR	95% CI	*P*-value	AOR	95% CI	*P*-value	AOR	95% CI	*P*-value	AOR	95% CI	*P*-value
Male gender	1.44	0.91–2.28	0.119	0.74	0.48–1.14	0.170	0.74	0.55–0.98	**0.035**	1.18	0.90–1.54	0.241	1.25	0.96–1.64	0.100
Age (years)	1.01	1.00–1.02	0.083	1.01	1.00–1.03	**0.018**	1.00	0.99–1.01	0.714	0.98	0.98–0.99	** < 0.001**	0.99	0.98–0.99	** < 0.001**
Swedish background	0.68	0.38–1.21	0.185	0.83	0.48–1.45	0.514	0.95	0.66–1.37	0.799	0.85	0.61–1.19	0.349	0.85	0.61–1.20	0.364
College/University education	1.24	0.79–1.93	0.344	1.93	1.29–2.88	**0.001**	1.60	1.23–2.08	**0.001**	1.21	0.94–1.57	0.144	1.20	0.93–1.55	0.160
Income (SEK/month)															
< 20,000/Other^a^	Ref			Ref			Ref			Ref			Ref		
20,000–39,999	1.04	0.59–1.81	0.900	1.19	0.73–1.94	0.484	1.01	0.71–1.43	0.974	1.05	0.76–1.46	0.755	1.01	0.73–1.40	0.971
≥ 40,000	0.92	0.50–1.69	0.784	1.42	0.79–2.57	0.241	0.81	0.56–1.17	0.254	1.07	0.75–1.52	0.719	1.07	0.75–1.52	0.702
Living alone	0.91	0.56–1.48	0.715	0.89	0.57–1.39	0.599	0.88	0.65–1.20	0.412	0.98	0.73–1.32	0.904	1.05	0.79–1.40	0.746
Swedish Health Care Region															
Stockholm-Gotland	Ref			Ref			Ref			Ref			Ref		
Mid-Sweden	0.59	0.33–1.07	0.084	0.72	0.41–1.27	0.252	0.80	0.54–1.18	0.258	1.15	0.78–1.69	0.471	0.86	0.58–1.25	0.423
Southeast	2.00	0.80–5.01	0.137	2.28	0.94–5.54	0.068	1.93	1.12–3.32	**0.018**	1.59	0.97–2.59	0.064	1.23	0.75–2.01	0.405
South	1.37	0.68–2.79	0.378	1.01	0.53–1.95	0.970	0.78	0.51–1.18	0.237	1.44	0.96–2.16	0.074	1.17	0.78–1.74	0.447
West	1.26	0.67–2.38	0.471	1.09	0.58–2.03	0.797	1.06	0.71–1.58	0.784	0.98	0.67–1.44	0.937	0.96	0.66–1.41	0.852
North	1.20	0.50–2.88	0.683	2.02	0.80–5.11	0.138	1.17	0.69–1.97	0.563	1.29	0.80–2.09	0.295	1.15	0.72–1.83	0.565

The regression models included all variables in the table. Significant *P*-values are given in **bold**

*AOR* Adjusted odds ratio, *CI* Confidence interval, *Ref*. Reference category, *SEK* Swedish Krona (10,000 SEK ≈ €885)

^a^ Including *Don’t know* and *Does not want to disclose*

**Table 9:** Results from weighted adjusted logistic regression analyses of the association between demographic characteristics and awareness of hormone therapy, processed meat, low level of physical activity, red meat, and low intake of fruit and vegetables as risk factors for cancer

	Hormone therapy			Processed meat			Low level of physical activity			Red meat			Low intake of fruit and vegetables		
Variable	AOR	95% CI	*P*-value	AOR	95% CI	*P*-value	AOR	95% CI	*P*-value	AOR	95% CI	*P*-value	AOR	95% CI	*P*-value
Male gender	0.71	0.54–0.92	**0.010**	0.79	0.60–1.04	0.096	1.20	0.92–1.58	0.186	0.88	0.67–1.16	0.372	0.91	0.68–1.20	0.491
Age (years)	1.00	0.99–1.01	0.677	0.99	0.99–1.00	0.159	0.99	0.99–1.00	**0.039**	1.00	0.99–1.00	0.270	1.01	1.00–1.01	0.157
Swedish background	1.13	0.81–1.58	0.476	0.82	0.58–1.18	0.285	0.99	0.70–1.40	0.951	0.93	0.66–1.31	0.682	0.85	0.60–1.21	0.364
College/University education	1.37	1.07–1.76	**0.014**	2.05	1.59–2.63	** < 0.001**	1.66	1.29–2.13	** < 0.001**	2.30	1.78–2.98	** < 0.001**	1.57	1.21–2.03	**0.001**
Income (SEK/month)															
< 20,000/Other^a^	Ref			Ref			Ref			Ref			Ref		
20,000–39,999	1.47	1.06–2.04	**0.020**	1.18	0.85–1.66	0.326	1.20	0.86–1.68	0.273	1.06	0.76–1.50	0.718	1.16	0.82–1.64	0.413
≥ 40,000	1.72	1.22–2.44	**0.002**	1.23	0.86–1.76	0.250	1.02	0.72–1.45	0.906	1.06	0.74–1.52	0.744	1.09	0.76–1.57	0.631
Living alone	0.93	0.69–1.25	0.640	1.05	0.77–1.42	0.765	0.84	0.63–1.13	0.258	1.11	0.82–1.50	0.511	1.05	0.77–1.42	0.769
Swedish Health Care Region															
Stockholm-Gotland	Ref			Ref			Ref			Ref			Ref		
Mid-Sweden	0.96	0.65–1.40	0.828	0.69	0.47–1.02	0.065	0.88	0.60–1.27	0.487	0.61	0.42–0.89	**0.010**	0.92	0.64–1.32	0.638
Southeast	1.63	1.00–2.64	**0.048**	0.86	0.52–1.41	0.555	1.28	0.78–2.08	0.329	0.69	0.42–1.12	0.129	1.24	0.75–2.07	0.398
South	1.04	0.70–1.56	0.831	0.76	0.50–1.16	0.205	0.90	0.59–1.35	0.600	0.64	0.42–0.98	**0.041**	1.00	0.65–1.53	0.991
West	1.03	0.71–1.50	0.868	0.90	0.60–1.33	0.593	0.97	0.66–1.43	0.894	0.84	0.57–1.25	0.395	1.34	0.89–2.02	0.155
North	1.31	0.81–2.11	0.264	0.74	0.46–1.20	0.226	1.13	0.71–1.80	0.612	0.84	0.52–1.36	0.476	1.01	0.62–1.64	0.980

The regression models included all variables in the table. Significant *P*-values are given in **bold**

*AOR* Adjusted odds ratio, *CI* Confidence interval, *Ref*. Reference category, *SEK* Swedish Krona (10,000 SEK ≈ €885)

^a^ Including *Don’t know* and *Does not want to disclose*

**Table 10:** Results from weighted adjusted logistic regression analyses of the association between demographic characteristics and awareness of low fibre intake, viral and bacterial infections, sedentary, low intake of whole grains, and not breastfeeding one’s child as risk factors for cancer

	Low fibre intake			Viral and bacterial infections			Sedentary			Low intake of whole grains			Not breastfeeding one’s child		
Variable	AOR	95% CI	*P*-value	AOR	95% CI	*P*-value	AOR	95% CI	*P*-value	AOR	95% CI	*P*-value	AOR	95% CI	*P*-value
Male gender	0.80	0.61–1.05	0.101	1.30	0.98–1.72	0.071	1.08	0.82–1.41	0.583	0.69	0.52–0.93	**0.015**	1.11	0.73–1.67	0.634
Age (years)	1.02	1.01–1.03	** < 0.001**	0.99	0.99–1.00	0.187	1.00	0.99–1.01	0.610	1.01	1.00–1.02	**0.003**	1.00	0.98–1.01	0.514
Swedish background	0.89	0.62–1.27	0.521	0.99	0.69–1.43	0.965	0.70	0.49–0.98	**0.039**	0.99	0.66–1.47	0.944	0.76	0.45–1.28	0.298
College/University education	1.99	1.52–2.60	** < 0.001**	1.60	1.22–2.11	**0.001**	1.49	1.16–1.92	**0.002**	1.58	1.19–2.11	**0.002**	1.67	1.11–2.52	**0.014**
Income (SEK/month)															
< 20,000/Other^a^	Ref			Ref			Ref			Ref			Ref		
20,000–39,999	1.28	0.91–1.81	0.154	1.26	0.88–1.81	0.204	1.19	0.85–1.65	0.310	1.09	0.75–1.58	0.653	1.27	0.70–2.30	0.430
≥ 40,000	1.35	0.95–1.93	0.093	1.43	0.99–2.06	0.057	1.07	0.75–1.52	0.706	1.32	0.90–1.93	0.159	1.37	0.77–2.42	0.283
Living alone	1.21	0.89–1.67	0.229	1.38	1.00–1.89	**0.049**	0.82	0.61–1.09	0.172	1.25	0.89–1.77	0.203	0.88	0.54–1.43	0.606
Swedish Health Care Region															
Stockholm-Gotland	Ref			Ref			Ref			Ref			Ref		
Mid-Sweden	0.96	0.67–1.37	0.813	1.00	0.67–1.50	0.987	1.10	0.76–1.60	0.621	1.16	0.78–1.70	0.463	1.42	0.80–2.54	0.235
Southeast	1.76	1.07–2.91	**0.026**	1.33	0.79–2.26	0.285	1.30	0.80–2.11	0.289	1.91	1.14–3.20	**0.014**	2.36	1.13–4.91	**0.022**
South	1.05	0.68–1.61	0.827	0.88	0.56–1.39	0.581	0.89	0.59–1.35	0.592	1.16	0.71–1.89	0.545	1.14	0.58–2.22	0.706
West	1.27	0.85–1.89	0.238	1.21	0.80–1.84	0.364	1.21	0.82–1.78	0.333	1.65	1.09–2.51	**0.019**	1.58	0.82–3.03	0.170
North	0.92	0.56–1.53	0.755	1.23	0.76–1.99	0.407	1.32	0.84–2.07	0.232	1.02	0.61–1.70	0.935	1.44	0.69–3.00	0.337

The regression models included all variables in the table. Significant *P*-values are given in **bold**

*AOR* Adjusted odds ratio, *CI* Confidence interval, *Ref*. Reference category, *SEK* Swedish Krona (10,000 SEK ≈ €885)

^a^ Including *Don’t know* and *Does not want to disclose*

Men were less likely to be aware of the following cancer risk factors; sunbeds, alcohol, hormone therapy and low intake of whole grains (compared to women). Older participants were more likely to be aware of the following cancer risk factors; radon, obesity, overweight, physical activity, low fibre intake and low intake of whole grains (Tables 7, 8, 9 and 10).

Moreover, participants with a Swedish background were less likely to be aware of sunbeds and a sedentary lifestyle as cancer risk factors (compared with participants with a foreign background). Participants that were living alone were 1.38 as likely to be aware of viral and bacterial infections as a risk factor for cancer, after adjusting for the other demographic characteristics (*P* = 0.049), compared with participants not living alone (Tables 7, 9 and 10).

Finally, we found significant differences between the HCRs related to the awareness of alcohol, hormone therapy, red meat, low fibre intake, low intake of whole grains and not breastfeeding as cancer risk factors, after adjusting for the other demographic characteristics (Tables 8, 9 and 10).

## Discussion

This cross-sectional study on cancer risk factors among the general public in Sweden found that awareness varies significantly between risk factors. Almost all respondents (97%) successfully identified smoking as a risk factor, whilst only 9% recognized that breastfeeding is a protective factor. Most respondents were aware that sun exposure, heredity factors, sunbeds and air pollution are risk factors. On the contrary, in descending order, low levels of awareness (< 50%) were found for physical inactivity (48%), hormone therapy (45%), red meat (39%), low intake of fruit and vegetables (33%), viral and bacterial infections (32%) and low intake of whole grains (24%).

### Results in context

Our results are in line with findings reported by other scholars. A study from Sweden and Denmark reports modest levels of public awareness on cancer risk factors [10]. Lagerlund et al. reported that the lowest levels of awareness among the Swedish participants were found for HPV-infections, low intake of fruits and vegetables and alcohol consumption [10]. It is noteworthy that the results from the former and the current study are similar, despite being conducted a decade apart, indicating that the public awareness has not improved.

Another recent study found limited awareness on the causal link between alcohol and cancer among almost 20 000 participants from 14 European countries. Only 53% of the participants knew that alcohol could cause cancer and even fewer (15%) reported that they knew that alcohol could cause female breast cancer [23].

In the context of cancer prevention, our findings, together with results reported by others [10, 23, 24], challenge the common perception that “everyone already knows what healthy lifestyle habits are”. In fact, for nine of the 20 established risk factors included in this study, awareness was below 50%. The low awareness of the cancer risk associated with low intake of whole grains, low levels of fruit and vegetables and sedentary behaviour is, from a broader public health perspective, particularly concerning. Especially since low intake of whole grains is among the leading contributors to non-communicable morbidity within the EU [25]. Moreover, dietary habits and physical inactivity are indirectly causally linked to overweight and obesity, which in turn are associated with several cancer types as well as other common noncommunicable diseases [18, 25].

In Sweden, unhealthy lifestyle habits are prevalent across groups with different socioeconomic positions [26]. There is, however, a social gradient across the whole cancer continuum, from prevention to mortality [27]. Disparities due to education level are prevalent for almost all cancer diagnoses. Vaccarella [28] found that across Europe, people with a shorter education had higher mortality rates for almost all cancer types, compared with people with a longer education.

This study found a discernible statistically significant educational gradient across nearly all risk factors, even when adjusting for confounding factors, indicating that individuals with higher education have greater awareness. Only for overweight, obesity, and air pollutions did the associations fail to reach statistical significance. These findings are particularly concerning, illuminating inequities in cancer prevention awareness in Sweden.

Being informed is a prerequisite for making well-informed health related decisions. Therefore, the inequity in cancer prevention awareness identified in this study is concerning. This has for example been studied in the adjacent field of cancer screening participation, where an educational gradient has been consistently observed across Europe (including Sweden) indicating lower participation rate among individuals with shorter education [29, 30]. Improving overall awareness, and particularly addressing disparities in awareness, may positively influence cancer prevention behaviours.

Previous research highlights the need for public information to be tailored to best fit the diverse needs and preferences of the population, in order to achieve effective outcomes [31]. Further, communicated information needs to be easy to understand, minimize stigmatization and guilt as well as being transparent and evidence-based, in order to support individuals [32–34]. Our results portray the importance of tailoring cancer prevention information to heterogeneous groups, to decrease inequities in awareness. In line with this, Wu et al. describe how advances in artificial intelligence (AI) may offer a strategy for enhancing awareness of cancer risk factors by supporting targeted, person‑centered communication strategies tailored to individual needs [35]. Moreover, research also suggests that mobile applications may serve as effective tools for increasing public awareness, but further studies are needed to strengthen the evidence base [36].

Furthermore, while others have reported the opposite [37], the results from this study showed that men (70%) were significantly more likely than women (58%) to agree that by changing lifestyle habits, people could reduce their cancer risk. To interpret this finding, it is important to consider how respondents may have understood the statement. Some participants may have perceived it as an evaluation of whether lifestyle factors matter when it comes to cancer risk, whereas others may have interpreted it as a statement about people’s capacity to successfully change their lifestyle. These distinct interpretations could have influenced response patterns.

Although not directly assessed in this study, the observed gender difference may be interpreted through the framework of locus of control (LoC). LoC refers to the extent to which individuals attribute life outcomes to internal versus external factors [38]. An external LoC denotes the belief that outcomes are primarily determined by forces beyond personal control, while a strong internal LoC reflects the perception that outcomes largely depend on one’s own actions and behaviours. Prior research has shown that men, on average, tend to score higher on measures of internal LoC [39, 40], suggesting that men may be more inclined to believe in the efficacy of personal agency and behavioural modification. At the same time, evidence indicates that women report greater cancer-related worry than men [41, 42]. This juxtaposition highlights the complexity of gendered patterns in cancer risk perception.

### Clinical implications and further research

The findings from this study provide actionable insights on how cancer prevention awareness may be strengthened. Even though effective prevention requires strategies that go well beyond awareness alone, our results underscore the continued importance of public health communication and awareness-raising initiatives. In particular, our findings highlight the critical need to prioritize efforts that promote equity. Addressing disparities in cancer prevention awareness requires targeted interventions reducing the educational gradient identified in this study. Such efforts are crucial for equitable access to cancer prevention information across diverse population groups.

To maximize the potential public health benefits of awareness initiatives, it is essential to prioritize those risk factors for which awareness levels are comparatively low. We see no justification for refraining from pursuing high levels of awareness across all cancer risk factors, despite their differing contributions to cancer incidence. Consequently, addressing current awareness gaps in cancer prevention among the Swedish general public requires system-level actions, with both public health and healthcare sectors playing central roles in supporting such efforts. However, promoting equity in cancer prevention awareness cannot be achieved through isolated efforts or by individual organizations. It requires long-term collaboration between different stakeholders and sectors of society.

Further research is needed to explore how cancer prevention information can be effectively tailored to meet the needs of diverse target audiences, particularly within an evolving communication landscape shaped by AI technologies, social media platforms and other emerging information sources.

### Strengths and limitations

Few studies have previously examined awareness of cancer risk factors among the general public in Sweden. Thus, this study adds important information to the scientific field. Additional strengths are the large and representative sample, as well as the use of post-stratifications weights in the statistical analyses. However, there are some limitations that need to be considered. Firstly, our results only provide a snapshot of cancer prevention awareness; more research and repeated surveys are needed in order to follow trends over time. This is particularly relevant since at the time of data collection, a nationwide public campaign aiming at eliminating cervical cancer in Sweden through human papillomavirus (HPV) vaccination took place. It is therefore possible that public awareness of viral and bacterial infections, such as HPV, as cancer risk factors is higher than prior to the campaign. Secondly, as mentioned in Hultstrand et al. [6], our results may inherent some selection bias since only Swedish-speaking persons with digital access have completed the questionnaire.

## Conclusions

Besides significant awareness gaps among the Swedish general public regarding several established cancer risk factors, this study found an educational gradient for most risk factors, illuminating important differences in cancer prevention awareness. Achieving meaningful improvements requires coordinated system-level and policy-level actions to reduce the educational gradient and ensure equitable access to information. This could, in turn, increase people’s abilities to make well-informed decisions about their lifestyle habits and preventive measures.

## Data Availability

The datasets used and analysed during the current study are available from the corresponding author upon reasonable request.

## Abbreviations

AOR: Adjusted odds ratio
ECAC: European code against cancer
EU: European Union
HCR: Health care region
HPV: Human papillomavirus
LoC: Locus of control
SD: Standard deviation
OR: Odds ratio
